# Automated Generation of Three-Dimensional Complex Muscle Geometries for Use in Personalised Musculoskeletal Models

**DOI:** 10.1007/s10439-020-02490-4

**Published:** 2020-03-17

**Authors:** Luca Modenese, Josef Kohout

**Affiliations:** 1 Department of Civil and Environmental Engineering, Imperial College London, London, UK; 2 Faculty of Applied Sciences, University of West Bohemia, Pilsen, Czech Republic

**Keywords:** Skeletal muscle, Musculoskeletal geometry, Moment arms, Lower limb, Line of action

## Abstract

The geometrical representation of muscles in computational models of the musculoskeletal system typically consists of a series of line segments. These muscle anatomies are based on measurements from a limited number of cadaveric studies that recently have been used as atlases for creating subject-specific models from medical images, so potentially restricting the options for personalisation and assessment of muscle geometrical models. To overcome this methodological limitation, we propose a novel, completely automated technique that, from a surface geometry of a skeletal muscle and its attachment areas, can generate an arbitrary number of lines of action (fibres) composed by a user-defined number of straight-line segments. These fibres can be included in standard musculoskeletal models and used in biomechanical simulations. This methodology was applied to the surfaces of four muscles surrounding the hip joint (iliacus, psoas, gluteus maximus and gluteus medius), segmented on magnetic resonance imaging scans from a cadaveric dataset, for which highly discretised muscle representations were created and used to simulate functional tasks. The fibres’ moment arms were validated against measurements and models of the same muscles from the literature with promising outcomes. The proposed approach is expected to improve the anatomical representation of skeletal muscles in personalised biomechanical models and finite element applications.

**Electronic supplementary material:**

The online version of this article (10.1007/s10439-020-02490-4) contains supplementary material, which is available to authorized users.

## Introduction

Computational models of the musculoskeletal system have been used in a variety of contexts, from estimating contact forces on lower limb joints^31^,^46^ to simulating musculotendon contraction mechanisms in healthy and pathological individuals.^5^ Despite the popularity of these models, there are limited options to represent the muscle anatomy, and the available musculoskeletal models^4^,^14^,^16^,^36^ are built using geometrical data from few dissection studies.^9^,^10^,^26^,^57^ This limitation impacts the approaches to personalised medicine because musculotendon paths in subject-specific models are defined using registration methods^35^,^47^,^53^ or statistical shape models^61^ that map existing representations of the muscular system to personalised bone geometries. The modelling assumptions about muscle anatomy are therefore maintained across applications, potentially hindering personalization and predictive accuracy. Based on previous literature, the two main design aspects to consider when representing the anatomy of skeletal muscles in multibody models are (a) the number of elements included in the representation (muscle *discretization* level), also called “fibres” in the following, and (b) the geometrical complexity of each of these fibres’ path, determined by the number of straight line segments constituting the line of action.

The number of fibres required to ensure an accurate representation of a muscle’s mechanical action has been previously investigated based on the dimensionality of their attachment sites,^54^ with only one study focusing on the lower limb.^52^ In that study, where errors due to muscle discretization were computed at the bone attachments and not at joint centres as in musculoskeletal simulations, it was found that the required level of discretization depended on the individual anatomy and on the lower limb posture. In a sensitivity study, Xiao and Higginson^59^ observed that the number of fibres affected muscle force estimation, while Moissenet *et al*. reported more accurate estimates of contact force at the tibiofemoral joint^37^ and hip joint^38^ for higher muscle discretization. Recently, other studies confirmed that high discretization level of the muscle surrounding the hip joint is necessary to provide an accurate estimation of joint contact forces.^32^,^58^ The effect of muscle discretization on musculoskeletal force outputs remains however largely unexplored, due to the lack of methodologies to systematically vary the level of discretization of the muscle representation.

It is common practice to represent muscle geometry in musculoskeletal models by connecting the origin and attachment sites with a series of line segments, enhanced using via points and wrapping surfaces that prevent bone penetration and improve bio-fidelity resulting in series-of-line-segments paths^3^,^20^ (referred to as *straight-lines approach* in the following). From the mechanical point of view, this is a valid representation of a three-dimensional muscle only as long as the line segments pass through the centroids of the force distribution in the considered muscle sections.^2^ This approach is therefore reasonable for muscles presenting fusiform shapes and well-defined muscle attachments, but less appropriate for those with complex paths and large attachment areas. To overcome this limitation, Jansen and Davy^24^ proposed a geometrical representation based on the line connecting the muscle section centroids (centroidal approach), which required a line of action with around 15 line-segments. They found that estimated moment arms were larger compared to those of the straight-lines approach, with differences up to 50% on certain components of the generated muscle moment. Moreover, their reported moment arms correlated with muscle volume, so highlighting the importance of considering individual muscle morphology. The lines of action’s geometrical representation can influence the predictions of musculoskeletal models; Modenese *et al*.^34^ suggested that the straight-lines representation of muscles surrounding the hip joint was limiting the accuracy of hip contact force predictions, while non-negligible differences between modelled and experimentally measured musculotendon lengths, that can influence muscle force generation, were reported both for lower and upper limbs models.^25^,^33^ Despite these known limitations, no approach has been developed to generate lines of action of appropriate complexity based on muscle morphological data, e.g. segmented muscle surfaces, to include them in musculoskeletal models.

In previous studies, continuous models provided realistic anatomical representations of skeletal muscles. Blemker and Delp^6^ developed a finite element model of hip muscles by mapping templates of fibre arrangements on surface meshes. The muscle geometries in their study were segmented from magnetic resonance imaging (MRI) scans collected on a young individual, and the deformations predicted by their model were validated against additional segmentations of MRI scans for multiple hip joint positions. The model was computationally expensive (5–10 CPU hours in 2005) and, although it produced fibre paths from which lengths and moment arms could be computed, those results were not employed in a multibody musculoskeletal model. Oberhofer *et al*.^41^ used the free form deformation technique to simulate a gait cycle with a model including deformable lower limb muscles, the shape of which was also validated against MRI scans, but no quantitative biomechanical variables were discussed. Kohout *et al*. developed a technique to decompose a muscle surface mesh in an arbitrary number of fibres and used it to create a simulation of walking intended as a visual aid for clinicians^27^,^28^ but did not provide any quantity of biomechanical interest. Despite the minimum computational cost, this approach^27^ still required an underlying straight-lines musculoskeletal model for solving the fibre kinematics. Other approaches to produce fibres from muscle surfaces are available in the literature,^12^,^23^,^30^,^42^ but it is unclear how to couple them with a multibody model for further biomechanical analyses.

The aim of this paper is to present an automated approach to generate muscle fibres based on surface meshes obtainable from medical images by segmentation. From each mesh, this method can produce an arbitrary number of fibres composed by a user-defined number of straight-line segments, usable as musculotendon actuators in musculoskeletal models and in biomechanical analyses. The approach is demonstrated on a hip joint model including highly discretised muscle representations of the surrounding muscles, for which moment arms will be computed using standard musculoskeletal approaches. The results will be assessed against a model with straight-lines muscle representations, created from the same anatomy, and results from previous literature, including a validated finite element model.^6^

## Materials and Methods

### Anatomical Dataset

**Figure 1:**
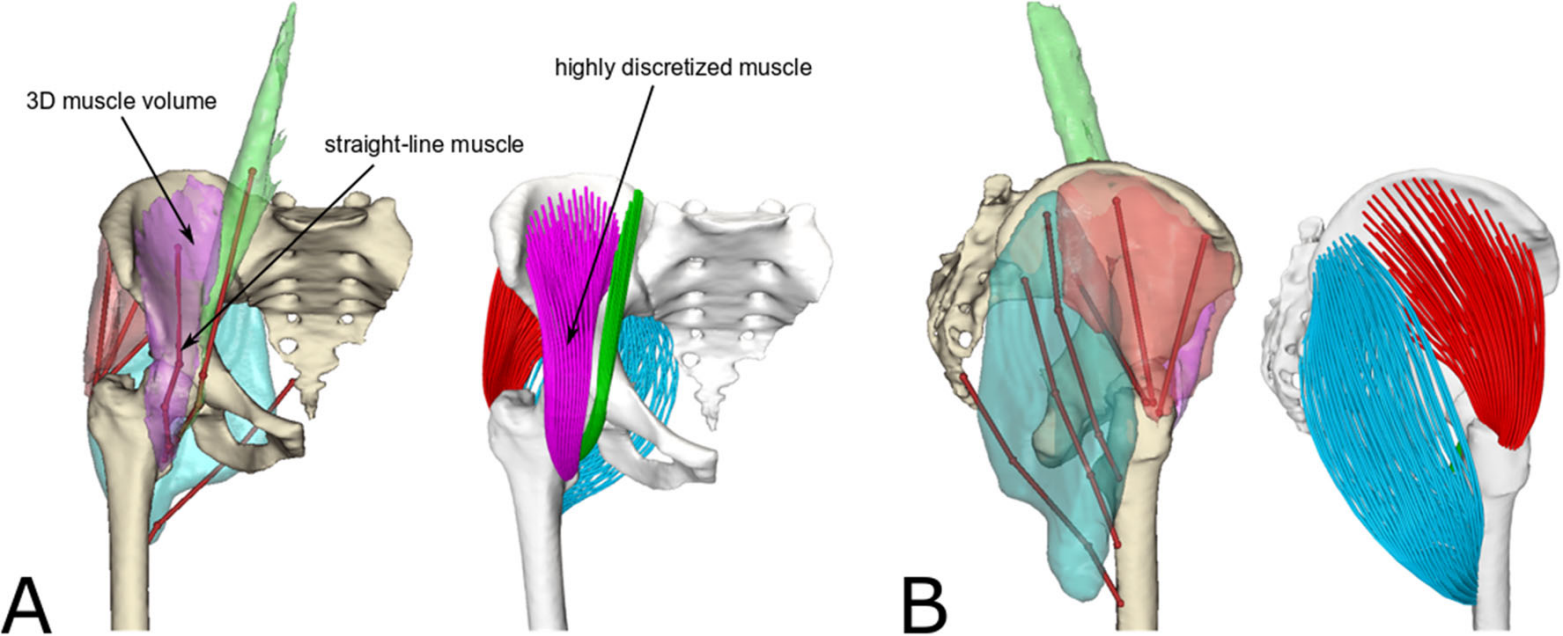
Frontal (a) and side view (b) of the bone and muscle geometries (*iliacus*: purple, *psoas*: green, *gluteus maximus*: cyan, *gluteus medius*: red) used for creating the musculoskeletal models. The straight-lines muscle representations and the segmented muscle surface meshes are shown together for comparison. The model with highly discretised muscle representations is shown on the right. All muscles were discretised using 100 fibres, each one consisting of a 15 line-segments polyline. Please note that although the *gluteus maximus* surface does not touch the femur, its insertion area lies on the bone.

A comprehensive anatomical dataset collected on a female cadaver (81 years old, 167 cm, 63 kg) was employed to create the musculoskeletal models used in this investigation (Fig. 1). The dataset, publicly available and known as LHDL dataset,^56^ was selected because it includes surface meshes of bones and muscles, segmented from computed tomography and MRI scans respectively, of quality similar to *in vivo* datasets. Muscle attachment areas were also identified and digitised during the dissection.^55^ The triangular muscle meshes were improved in a pre-processing step by removing non-manifold edges, duplicated vertices and degenerate triangles, followed by smoothing using MeshLab.^13^

### Musculoskeletal Models

The geometries of the pelvis and right femur were employed in NMSBuilder^51^ to create a skeletal model of the right hip joint (Fig. 1), represented as a three-degrees of freedom ball-and-socket joint centred by fitting a sphere to the femoral head surface. This kinematic model was then exported in OpenSim 3.3 format^15^ and used as common baseline for both models described below.

#### Model with Highly Discretised Muscle Representations

Each muscle surface mesh was processed in two stages to generate a set of muscle fibres used in the simulations: (1) a *muscle geometry decomposition* step, performed in the scanning pose, in which the mesh is transformed in a user-defined number of fibres, and (2) a *fibre kinematic* step, in which the geometry of the fibres from the first step is updated to a new skeletal pose.

##### Muscle Geometry Decomposition

As the algorithm employed in the muscle geometry decomposition is described in details in a previous publication,^28^ only an overview of its main steps will be presented here.

**Figure 2:**
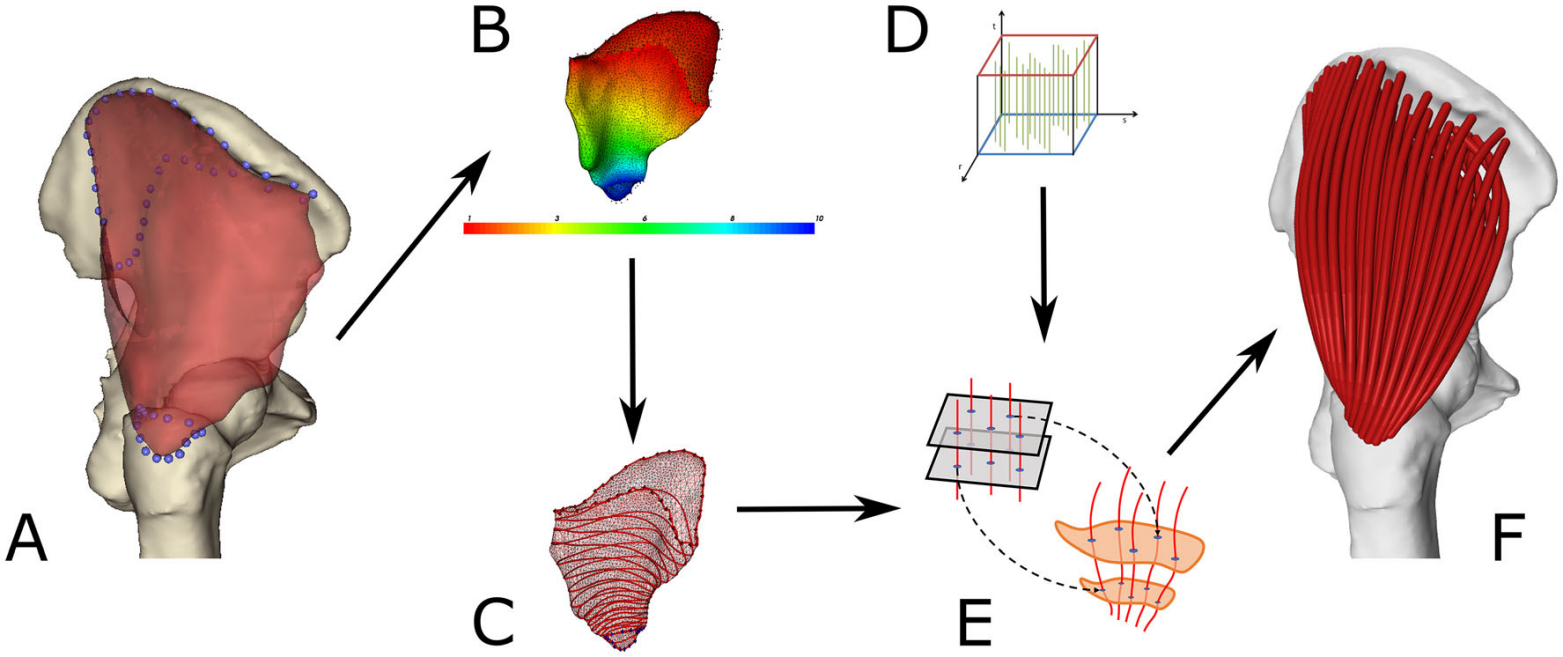
Sequence of operations used for decomposing a muscle volume mesh in an arbitrary number of fibres of user-defined resolution. A muscle surface mesh, in red, and attachment areas, outlined by the blue markers, are taken as input (a). The attachment sites are mapped on the muscle mesh, from which their projected area is removed. A scalar field is defined on the resulting surface (b) and as many isolines as the desired fibre points (c) are identified. A muscle architecture template (d), containing the number of fibres desired from the decomposition, is then mapped to the muscle mesh using planes corresponding to the isolines (e), so generating fibres that can be imported in a standard OpenSim model (f). In this example, the *gluteus medius* is discretised in 100 fibres, each one consisting of 15 straight-line segments.

The required inputs of the method (Figs. 2a and 2d) are (1) a triangular surface mesh representing the muscle geometry, (2) a fibre template providing geometrical information about the internal fibre arrangement of the muscle and (3) the attachment areas of the muscle (origin and insertion), described as sets of landmarks fixed on the bone.

Firstly, the attachment areas are projected from the bones to the muscle mesh, outlining two areas that are subsequently removed, to produce a surface with two boundaries. A piece-wise linear scalar field, presenting minimum value on the origin boundary and maximum on the insertion one, is then computed over the vertices of the mesh (Fig. 2b).^17^ Contours corresponding to field isolines, i.e. lines connecting points where the field value is constant, are extracted for as many values as required by the user-specified number of straight-line segments in each fibre (Fig. 2c).

To represent muscle fibre architecture, templates consisting of unit space with an arbitrary (user-defined) number of fibres connecting two attachment areas were employed, similarly to Blemker and Delp.^6^ The fibres are expressed analytically by Bezier spline curves. In the decomposition step, an appropriate template is selected and evenly sliced using as many parallel planes as the specified fibre points. The resulting template sections are then mapped one-to-one to the contours of the scalar field isolines (Fig. 2e). As the position of the fibres is expressed relative to the contour of the template using generalized barycentric coordinates,^22^ they can be mapped on the muscle mesh using the same transformation. The fibres’ geometry is finalised with a step that ensures they connect with the attachments followed by a quadratic smoothing to eliminate noise (Fig. 2f).

**Figure 3:**
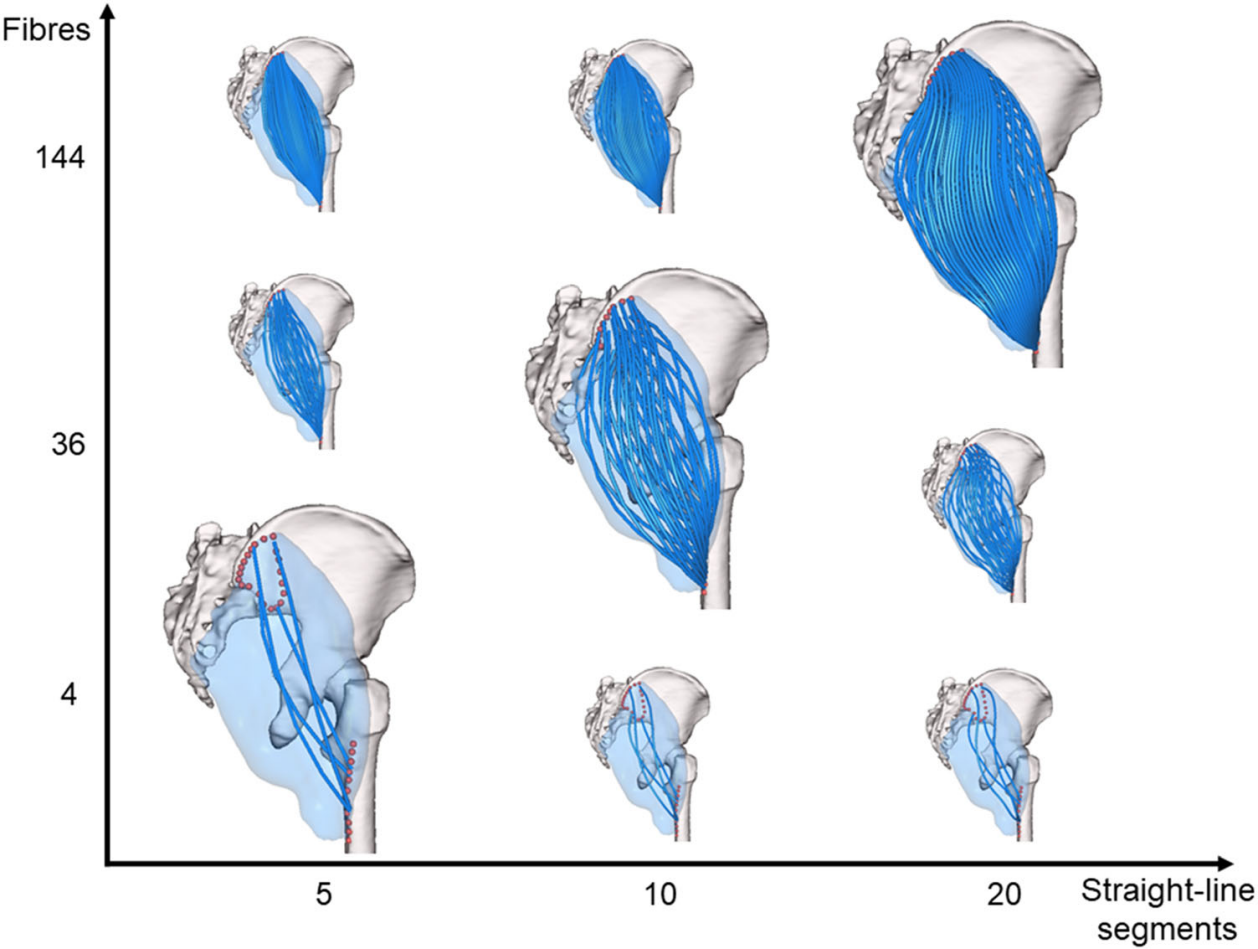
Muscle representations obtainable for *gluteus maximus* by combining different levels of muscle discretization (from 4 to 144 fibres) and numbers of line segments in the fibres (from 5 to 20 line-segments per fibre).

The muscle geometry representation resulting from this workflow can be customized by the user by choosing the total number of fibres and straight-line segments per fibre (Fig. 3).

The surface meshes of *gluteus maximus*, *gluteus medius*, *iliacus* and *psoas* were decomposed in highly discretised models (100 fibres) using a template with parallel fibres composed by 15 line-segments, similarly to the centroidal paths of Jensen and Davy^24^ (Fig. 1). This was done in the MuscleWrapping software, part of a larger LHPBuilder application developed within the VPHOP project^1^, now concluded. In this explorative study, the pelvic attachment area of *iliacus* was used as origin also for the *psoas* muscle to avoid modelling its multiple origins on the lumbar spine. These muscles were selected because of their complex geometry and for consistency with Blemker and Delp.^6^

##### Fibre Kinematics

The algorithm solving the kinematics of the produced fibres is based on binding the points of the fibres to the bones using an automated procedure. As justified in details in the supplementary materials, every fibre point $i$ was associated with its two nearest bones, and its kinematic position $V^{\prime}_{i}$ calculated as a linear combination of the transformations of its rest-pose position $V_{i}$ with respect to these bones as:

$$V^{\prime}_{i} = \mathop \sum \limits_{j = 1}^{2} w_{ij} \cdot \left[ {\begin{array}{*{20}c} {R_{j} } & {T_{j} } \\ \end{array} } \right] \cdot V_{i} \quad i = 1 \ldots n$$

where $n$ is the number of points in the fibres, $R_{j}$ and $T_{j}$ are the rotational and translational transformations of the *j*-th nearest bone and $w_{ij} \in 0,1$ is a blending weight determining the bone influence, with $\sum\nolimits_{j = 1}^{2} {w_{ij} } = 1$. Although other options exists (see supplementary materials), the weights $w_{i1}$ in this study were computed from the relative position $t = {{\left( {i - 1} \right)} \mathord{\left/ {\vphantom {{\left( {i - 1} \right)} {\left( {n - 1} \right)}}} \right. \kern-0pt} {\left( {n - 1} \right)}}$ of the *i*-th fibre point $V_{i}$ on the fibre (measured from the fibre origin $V_{1}$) using a quadratic function $f\left( t \right)$:

$$w_{i1} = f\left( t \right) = a \cdot t^{2} + b \cdot t + c;\quad w_{i2} = 1 - w_{i1} ;$$

where $a$, $b$, and $c$ are muscle-specific parameters that determine how quickly the influence of an attachment bone diminishes along the fibre length. The first and the last fibre point positions are governed by the pelvis and the femur only, i.e., $f\left( 0 \right) = 1$ and $f\left( 1 \right) = 0$, implying that $c = 1$ and $b = - \left( {a + 1} \right)$, thus leading to a formula with just one muscle-specific parameter $a$ to specify. In the current simulations, the value of $a$ was determined for each muscle by trial and error aiming to visually minimize the muscle-bone penetrations throughtout the flexion/extension range of motion (*psoas*: − 0.042, *iliacus*: − 0.024, *gluteus maximus*: − 0.042, *gluteus medius*: 0.0).

#### Model with Straight-Lines Muscle Representations

Musculotendon paths of *iliacus*, *psoas*, *gluteus medius* and *gluteus maximus* were defined as in Modenese *et al*.^35^ using the straight-lines muscle representation of the popular model *gait2392*^16^ as reference atlas, with minor manual adjustments to take advantage of the available muscle geometries (Fig. 1). Consistently with the *gait2392* model, *iliacus* and *psoas* were modelled using a single line of action, while *gluteus medius* and *gluteus maximus* were discretized using three lines of action each.

### Simulations and Validations of Muscle Moment Arms

Simulations of hip extension/flexion (− 10° to 60°), hip ab/adduction (− 40° to 40°) and hip internal/external rotation (− 30° to 30°), performed in steps of 2°, were generated using both models.

The geometry of the fibres in the model with highly discretised muscles was updated at each frame of the kinematics using the application programming interface (API) of OpenSim v3.3^15^ from MATLAB R2017b. The length $l$ of each fibre was then computed through the same API, interpolated with a 4th order polynomial function and used to calculate the moment arm $r_{i,j}$ of the *i-th* fiber with respect to the *j*-th coordinate using the tendon excursion method^1^:

$$r_{i,j} = \frac{{\partial l_{i} }}{{\partial \theta_{j} }}$$

The model with straight-lines muscles, instead, was imported in OpenSim 3.3^15^ and its standard *MuscleAnalysis* tool was used to compute the moment arms^49^ for the same hip joint tasks. This approach was preferred to the tendon excursion method because it is more accurate for lines of action including conditional via points, for which the moment arm can change non smoothly (*iliacus* and *psoas* at around 40° flexion).

The moment arms computed with the highly discretised muscles were compared at each hip joint pose against those of the model with straight-lines muscles and data available from previous literature,^3^,^18^,^40^ including the results of the validated finite element model of Blemker and Delp,^6^ which were digitised using Graph Grabber v2.0 (https://www.quintessa.org). The percentage of poses for which the moment arms were in agreement, i.e. for which the range calculated with the highly discretized muscles model included the values from the other model or measurement, was calculated and reported. The peak and mean values of moment arms across the entire range of motion from the highly discretised muscles, together with those from the straight-line muscles and an estimation from Blemker and Delp^6^ were also reported for each task.

## Results

For all muscles, the decomposition step took around 20 ms, while the simulations with highly discretised muscle representations took around one minute on a Z640 Dell Workstation (RAM: 64 GB, CPU: E5-2630 2.40 GHz).

**Figure 4:**
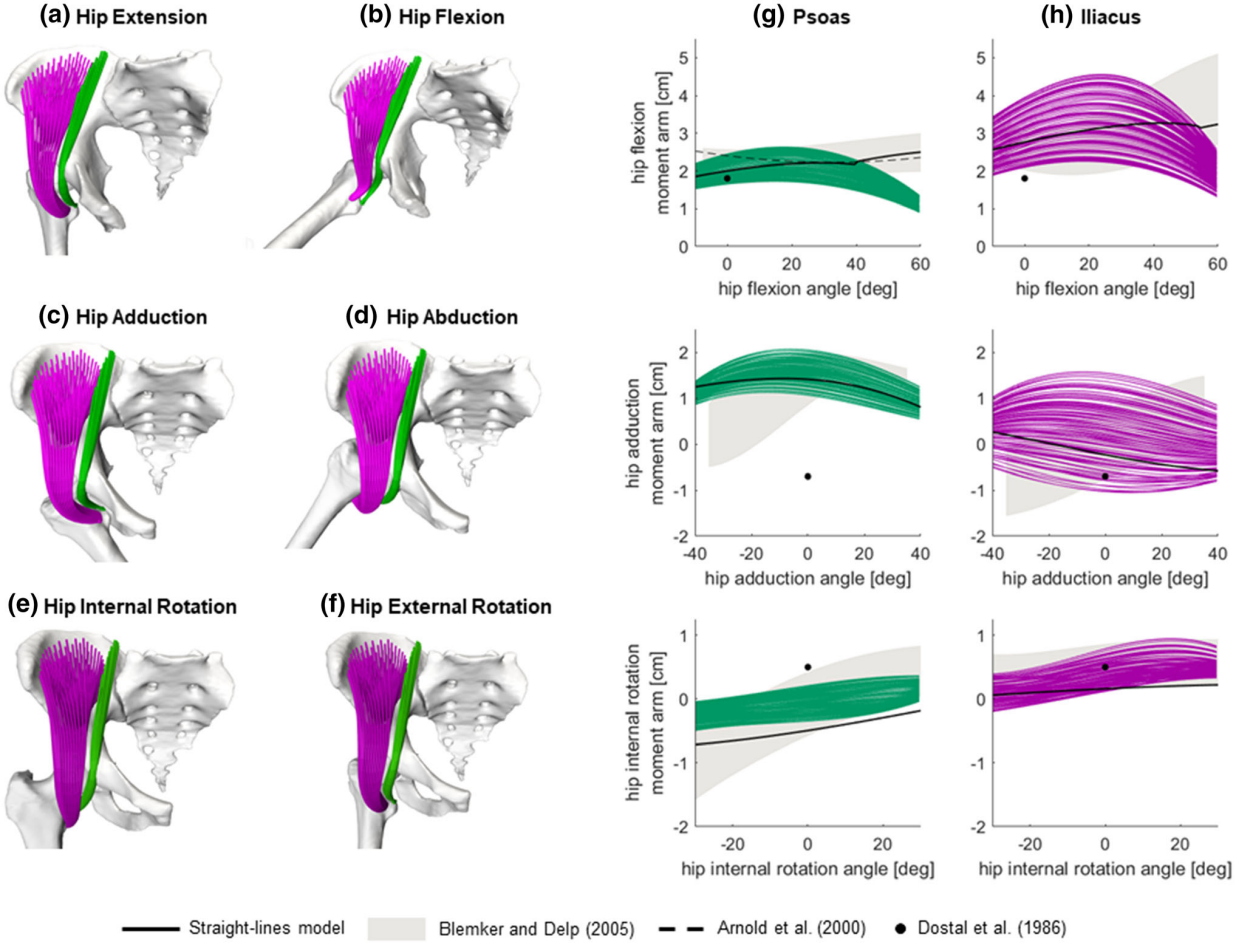
Results of the simulations of hip functional tasks for the *iliacus* and *psoas* muscles (in purple and green respectively). The resulting geometries of the highly discretised muscle models are shown on the left (a–f), and the correspondent moment arms are presented, for all fibres, on the right (g–h). The resulting moment arms are compared against the moment arms of the model with straight-lines muscles and other studies from the literature (see legend).

Overall, the results of the simulations were visually realistic for *psoas* and *iliacus* (Figs. 4a–4f), with the exception of flexion angles larger than 40 degrees (Fig. 4b), for which some fibres, especially of *psoas*, were penetrating the pelvis ridge geometry. On average, the moment arms from the straight-lines muscles were within the range estimated by the highly discretized muscles in 57% of the hip joint poses for *psoas* and 83% for *iliacus* (Table 1). Compared to Blemker and Delp,^6^ some differences in trend were observed for flexion angles larger than 35 degrees for both muscles, and when simulating ab/adduction for *iliacus* (Figs. 3g, 3h), leading to slightly lower level of agreement (*psoas*: 50%, *iliacus*: 77%).

**Table 1:** Comparison of the moment arms calculated with highly discretized muscles against estimations from the straight-lines muscles and previous data available in the literature

Considered model or study	Description of metrics	Hip task	Psoas	Iliacus	Gluteus maximus	Gluteus medius
Straight-lines muscles	Percentage of poses in which moment arms are within the range estimated by the model with highly discretized muscles.	Flexion/extension	72%	90%	91%^a^	99%^a^
		Adduction/abduction	100%	100%	77%^a^	79%^a^
		Internal/external rotation	0%	60%	78%^a^	85%^a^
		Mean	57%	83%	82%	88%
Blemker and Delp^6^	Percentage of area outlined by results from Blemker and Delp^6^ that is overlapping with the area outlined by the highly-discretized moment arms results	Flexion/extension	62%	71%	65%	81%
		Adduction/abduction	44%	79%	86%	54%
		Internal/external rotation	44%	79%	86%	95%
		Mean	50%	77%	79%	77%
Dostal *et al*.^18^	Reported values fall within the range estimated by the model with highly discretized muscles (Y/N: yes/no)	Flexion/extension	Y	N	Y	Y (72%^b^)
		Adduction/abduction	N	Y	Y	Y
		Internal/external rotation	N	Y	Y	Y
Nemeth and Ohlsen (1985)	Reported values fall within the range estimated by the model with highly discretized muscles (Y/N: yes/no)	Flexion/extension	–	–	Y (91%^b^)	–
		Adduction/abduction	–	–	Y	Y
Arnold *et al*.^3^	Percentage of poses in which moment arms are within the range estimated by the model with highly discretized muscles	Flexion/extension	61%	–	–	–

^a^Averaged across the three fibres representing this muscle in the straight-lines model

^b^Percentage estimated as for the model with straight-lines muscles

**Figure 5:**
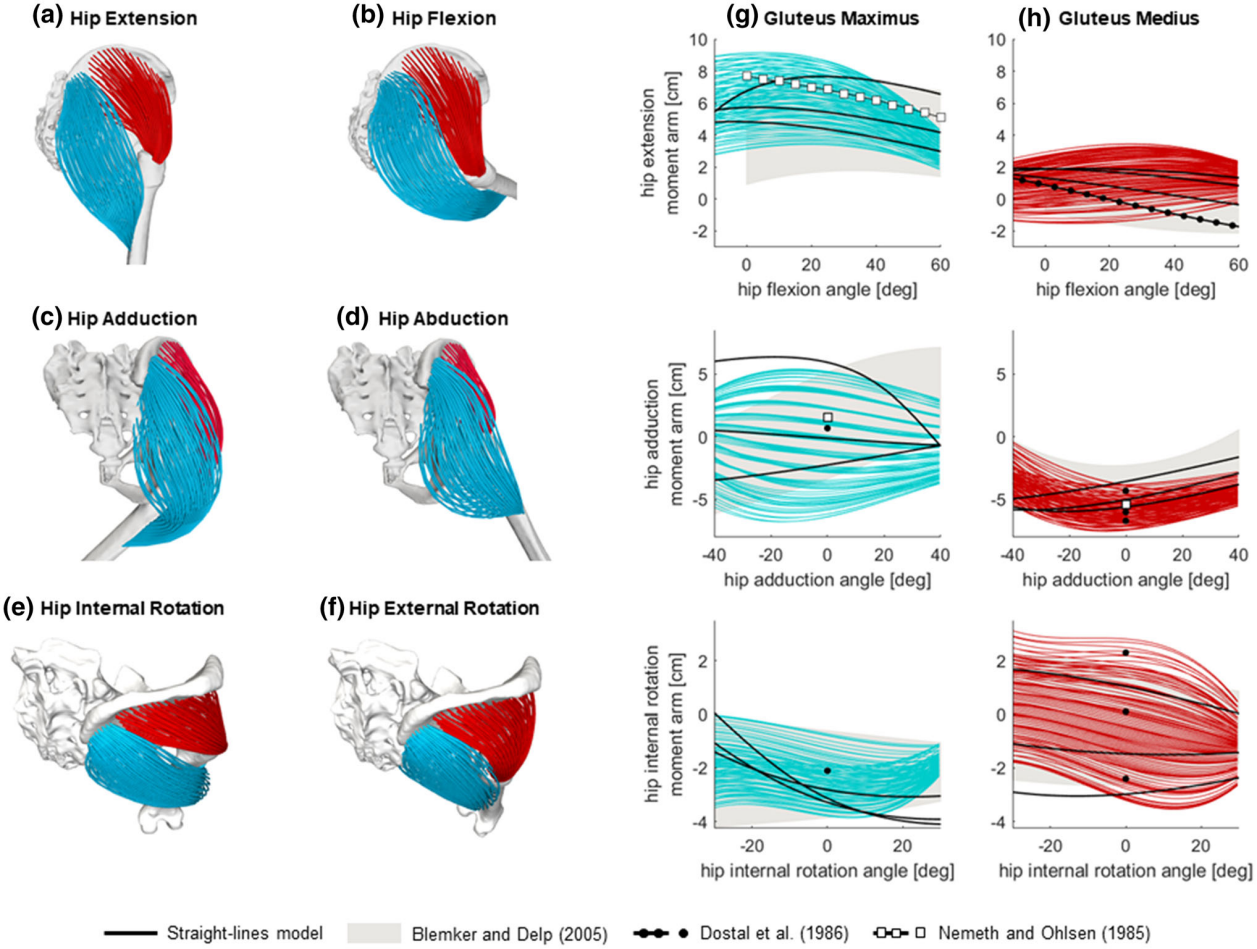
Results of the simulations of hip functional tasks for the *gluteus maximus* and *gluteus medius* muscles (in cyan and red respectively). The resulting geometries of the highly discretised muscle models are shown on the left (A-F), and the corresponding moment arms are presented, for all fibres, on the right (g–h). The resulting moment arms are compared against the moment arms of the model with straight-lines muscles and other studies from the literature (see legend).

The simulations of *gluteus maximus* and *gluteus medius* also generated visually satisfactory geometries (Figs. 5a–5f), with fibres of the former muscle minimally penetrating the ischium and sacrum bones towards the extreme joint angles of hip flexion and adduction respectively, e.g. Figs. 5c. The moment arms of the straight-lines muscles model were consistent with those of the highly discretized model on average in 82% of the considered poses for gluteus maximus and 88% for gluteus medius (Figs. 5g, 5h, Table 1). The comparison with Blemker and Delp^6^ also suggested a remarkable similarity of the estimated moment arms (gluteus maximus: 79%, gluteus medius: 77%).

Moment arms from a physical model^18^ and medical images^40^ were also in strong agreement with those of the highly discretized muscles (in 9 out of 12 comparisons and three out of three comparisons respectively, see Table 1). Reasonable consistency, on 61% of the considered hip flexion angles, was also found with the cadaveric measurements of Arnold *et al*.^3^

The upper and lower bounds of the moment arms computed from the OpenSim models, and those from the digitised results of Blemker and Delp,^6^ are reported for all muscles in Table 2. The peaks and means of moment arms were consistent among the three models for the majority of the considered hip joint poses.

**Table 2:** Peak values (minimum and maximum) and mean values with standard deviations (sd) of moment arms calculated across the considered ranges of motion for the highly discretized muscles, the straight-lines muscles and the results extrapolated from Figs. 6 and 7 of Blemker and Delp^6^ using Graph Grabber 2.0

Hip task	Model	Muscle moment arms (cm)											
		Psoas			Iliacus			Gluteus maximus			Gluteus medius		
		Min	Max	Mean (sd)	Min	Max	Mean (sd)	Min	Max	Mean (sd)	Min	Max	Mean (sd)
Flexion(+) /extension (−)	Highly discretized muscles	0.9	2.6	1.9 (0.3)	1.3	4.6	3.0 (0.4)	− 9.2	− 1.9	− 5.7 (0.9)	− 3.5	1.5	− 1.5 (0.3)
	Straight-lines muscles	1.8	2.5	2.2 (0.2)	2.6	3.3	3.1 (0.2)	− 7.7	− 2.9	− 5.5 (1.5)	− 1.9	0.4	− 1.3 (0.6)
	Blemker and Delp (2005)	1.7	3.0	2.3 (0.1)	1.9	5.1	3.0 (0.4)	− 7.2	− 0.9	− 4.2 (0.3)	− 1.7	2.1	− 0.0 (0.3)
Adduction (+)/abduction (−)	Highly discretized muscles	0.5	2.1	1.4 (0.2)	− 1.0	1.6	0.2 (0.2)	− 6.8	5.4	− 0.6 (0.3)	− 7.5	− 0.4	− 5.1 (0.6)
	Straight-lines muscles	0.8	1.4	1.3 (0.2)	− 0.6	0.3	− 0.2 (0.3)	− 3.5	6.4	0.8 (3.6)	− 5.9	− 1.6	− 4.5 (1.0)
	Blemker and Delp 6	− 0.5	2.0	1.2 (0.5)	− 1.5	1.5	− 0.0 (0.5)	− 6.1	7.1	0.8 (1.9)	− 5.4	0.6	− 3.1 (0.7)
Internal (+)/external (−) rotation	Highly discretized muscles	− 0.5	0.4	− 0.1 (0.1)	− 0.2	0.9	0.3 (0.1)	− 3.9	0.0	− 2.3 (0.3)	− 3.5	3.1	− 0.4 (0.5)
	Straight-lines muscles	− 0.7	− 0.2	− 0.5 (0.2)	0.1	0.2	0.2 (0.0)	− 4.1	0.1	− 2.8 (0.2)	− 3.0	1.7	− 1.1 (1.9)
	Blemker and Delp 6	− 1.8	0.8	− 0.2 (0.4)	− 0.1	0.9	0.5 (0.1)	− 4.2	− 0.1	− 2.2 (0.0)	− 2.7	2.2	− 0.4 (0.2)

Please note that the mean values for Blemker and Delp^6^ were computed using the digitised upper and lower boundaries of the results and that, differently from Fig. 5, here hip extension is negative in sign

## Discussion

The aim of this investigation was to present an automated technique to create complex, three-dimensional representations of skeletal muscles from their surface meshes that can be used in standard musculoskeletal models and demonstrate its use on a hip joint model. The proposed approach overcomes the traditional dualism of straight-lines versus more geometrically complex muscle representations such as centroidal lines, because paths of varying complexity can be automatically generated based on the user’s specifications. At the same time, the muscle discretization level can be altered in a systematic, reproducible way while still ensuring anatomical accuracy of the fibre set (Fig. 3).

In the absence of additional MRI scans for validating the highly discretised muscle geometries in various poses, we created a musculoskeletal model with straight-lines muscles, as this is the state of the art for representing musculotendon anatomies in multibody approaches. Results were also evaluated against a validated finite element model^6^ and previous measurements of moment arms from medical images and physical models.^3^,^18^,^40^ The agreement with the straight-lines muscles model and previous studies was generally very positive, especially for the hip extensors (Figs. 5g, 5h). For *psoas* and *iliacus*, however, we observed deviations from the straight-lines model for hip flexions larger than 40 and 55 degrees respectively. At those angles, conditional via points in the straight-lines muscles became inactive, similarly to the reference model *gait2392*, letting the moment arms increase consistently with experimental measurements^3^ and the results of the validated finite element model.^6^ Conversely, in the current formulation of the proposed technique the fibre points behaved essentially like via points with position regulated by a weight function but nevertheless active throughout the motion, so creating a sort of “adhesion” to the femur at high hip flexion (see Fig. 3b, in the hip joint area) that affected the moment arms estimation. We plan to improve this limitation of the methodology by implementing a position-based dynamics system^39^ that will have the further benefit of detecting muscle-bone contacts and preventing the occasional muscle-bone penetrations we observed. Further differences from Blemker and Delp^6^ could be attributed to various causes, including the identification of the muscle attachments, the muscle morphology in the elderly specimen (54 years older than their participant) and different segmentation of the *psoas* muscle, which presents in their study a planar cut at the level of the sacrum. Our results, however, compared overall positively against the validation datasets, suggesting that the new technique provides realistic muscle fibre configurations, especially within the range of motion typical of walking^48^ (hip extension/flexion: − 10° to 40°, ab/adduction: − 10° to 10° and axial rotation: − 7° to 7°).

The presented methodology presents some limitations. First, the approach requires muscle attachment areas, which are normally not available for *in vivo* datasets. They can, however, be estimated using statistical shape approaches^19^,^61^ or mapped from existing atlases, like the dataset provided with this paper, using registration techniques.^43^ Second, personalized surface meshes of muscles are also required by this technique, and they are currently time-consuming to segment from medical images. However, semi-automated segmentation procedures are becoming available both in commercial^29^ and open-source software,^60^ and, depending on the intended application, it might not be necessary to represent all the muscles as highly discretised, but only those of interest, e.g. presenting abnormal volumes that could affect moment arms.^21^ Third, only a template with parallel fibres was used in the current investigation, but the methodology is straightforward to extend^27^ to fibre templates proposed in previous studies.^6^,^7^

In conclusion, we believe that the proposed approach is a promising and fully automated solution to provide subject-specific representations of muscle geometries usable in multibody models that could benefit multiple applications in biomechanics. For example, sensitivity studies on muscle discretization similar to Valente *et al*.^52^ and their extension to muscle forces and joint reactions will be enabled. Future studies will investigate aspects of the methodology that require clarification before adoption in automated workflows for musculoskeletal and finite element simulations, such as sensitivity analyses with respect to the desirable number of straight-line segments in muscle fibres and to the uncertainty in muscle attachment areas identification, and in more advanced applications like the use of the computed moment arms and muscle lengths for muscle force estimation in dynamic simulations. Finite element models will particularly benefit from this technique because highly discretised muscle forces can be easily distributed on attachment areas, so avoiding stress concentrations on a small set of attachment nodes^45^ while still applying equilibrated force sets provided by the multibody systems.^44^,^50^ The muscle decomposition could also inform finite element models of skeletal muscles about fibre arrangements, or be replaced by experimentally derived fibre arrangements, e.g. from diffusion tensor imaging,^8^,^11^ to use in kinematic simulations.

The anatomical dataset and the OpenSim models used in this study are freely available for download at https://github.com/ComputationalBiomechanics/3d-muscles and https://simtk.org/projects/3d-muscles.

## Electronic supplementary material

Below is the link to the electronic supplementary material.

Supplementary material 1 (PDF 75 kb)

## Footnotes

1 https://cordis.europa.eu/project/rcn/87265/factsheet/en
