# Supplementary material for: Automated Generation of Three-Dimensional Complex Muscle Geometries for Use in Personalised Musculoskeletal Models
Source: Ann Biomed Eng. 2020 Mar 17;48(6):1793–804. doi: 10.1007/s10439-020-02490-4 (PMC7280327; doi:10.1007/s10439-020-02490-4)
Supplement: Supplementary file 1 — Supplementary material 1 (PDF 75 kb) [file 10439_2020_2490_MOESM1_ESM.pdf]

Supplementary materials

Appendix: Connecting the points of a muscle fibre to the moving bones

In traditional musculoskeletal models, each point of a muscle fibre is connected rigidly to a segment of the model, following the structure of a muscle representation atlas or the operator's design. In the presented high-resolution muscle representation, this would be unpractical, so an automated solution is necessary. The most straightforward approach for solving the fibre kinematics of the produced fibres is to bind the point to its nearest bone. Due to the complexity of the pelvic area, however, there might be a point whose nearest bone differs from the bone associated with its adjacent points, as illustrated in Figure A.1 (see points V_2, V_3, and V_4). This would result in a completely unrealistic geometrical path of the fibre. Even if no such point exists, the smoothness of the fibre could be severely impacted. For example, if V_3 were associated with the femur, the fibre line-segments V_1 to V_5 would form a smooth curve but the rest (V_5 to V_6) would be just a long straight line-segment, likely to penetrate the bones in extreme poses scenarios. This justifies the implementation of the proposed fibre kinematic approach based on weight functions, as preserving the integrity of the muscle paths while also ensuring their smoothness.

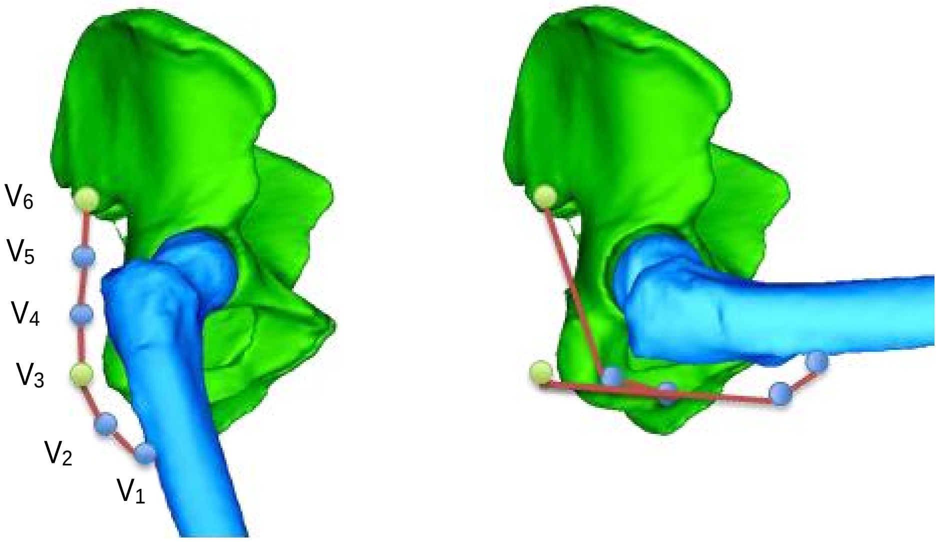

Figure A.1 Update of the fibre geometry using the common approach of musculoskeletal models that bind the via points, which the fibre passes through, to a single bone – in this case, automatically detected to be the nearest one.

It is possible to further justify also the choice of the weight function used in the fibre kinematics implementation. A popular choice[1] for setting the weights for skeletal animations in computer graphics is to set them proportional to the inverse Euclidean distance to the appropriate nearest bones using the formula:

$$w_{ij} = \left(\frac{\frac{1}{d_{ij}}}{\sum_{k=1}^{2} \frac{1}{d_{ik}}} \right)^m \rightarrow w_{i1} = \left(\frac{d_{i2}}{d_{i1} + d_{i2}} \right)^m ;\ w_{i2} = \left(\frac{d_{i1}}{d_{i1} + d_{i2}} \right)^m$$

In this formula, d_{ij} denotes the Euclidean distance between point V_i and its *j*-th nearest bone and m is a parameter determining how quickly the effect of the more distant bone should diminish. Most typically, this parameter is set to 2.

In musculoskeletal models, bones are quite different from those in skeletal animations (where they are just line segments connecting the joints) and, therefore, inverse Euclidean distance weights produce undesirable results in the complex pelvic area, no matter what value of the parameter m is. For example, the fibre point V_2 (see Figure A.1) would be strongly influenced by the pelvis segment even though it is very far from the muscle origin (on the pelvis) and much closer to the insertion (on the femur). The implemented function, on the other hand, uses a weighting function based on the relative distance between point V_i and the fibre origin V_1 that avoids this issue:

$$t = \frac{i-1}{n-1}; \qquad w_{i1} = a \cdot t^2 - (a+1) \cdot t + 1; \qquad w_{i2} = 1 - w_{i1};$$

where n is the number of points in the muscle fibres and a is the muscle-specific parameter with the default value being 0.

REFERENCES

1. Jacobson, A., Deng, Z., Kavan, L. Lewis, J.P. Skinning: real-time shape deformation. in ACM SIGGRAPH 2014 Courses. ACM. 2014.
